# The street space planning and design of artificial intelligence-assisted deep learning neural network in the Internet of Things

**DOI:** 10.1016/j.heliyon.2024.e35031

**Published:** 2024-07-22

**Authors:** Lei Song

**Affiliations:** School of Design, Shandong University of Arts, Jinan, 250300, China

**Keywords:** Street space, Green looking ratio, Fully convolutional network, Historic urban area

## Abstract

This study begins by discussing Internet of Things (IoT) technology and analyzing the classification of street space into four types, along with the Green Looking Ratio (GLR). Following this, the Fully Convolutional Network (FCN)-8s framework is employed to construct a street view image semantic segmentation model based on FCN principles. Subsequently, IoT technology is utilized to analyze the proportion of GLR and satisfaction in the street space within the historical urban area of T City. The findings reveal a significant positive correlation (significance level p < 0.05, R^2^ = 0.919) between the GLR satisfaction score of street view images and the average GLR of the area. Among the four types of street space—life leisure, historical streets, traffic areas, and landscape-style streets—the dissatisfaction rates with GLR are 35 %, 33 %, 20 %, and 18 %, respectively, correlating with varying GLR satisfaction levels. To enhance street space greening, planting ponds and boxes are proposed for "blind spots” and "dead corners,” thereby completing greenery in these areas. These initiatives aim to improve street greening policies, integrate street function zones, and advance the scientific greening of urban streets. The analysis of GLR and satisfaction in street spaces provides valuable insights for refining urban street space greening efforts.

## 1 Introduction

With the rapid urbanization in China, urban construction in the new era now focuses more on improving the quality of small and medium-sized living environments rather than extensive expansion and transformation. Streets, alleys, and other public spaces crucial to daily life and heavily used are being re-evaluated [1,2]. Historically, the planning, design, and management of urban road spaces have primarily prioritized motor vehicle traffic efficiency. This approach has often overlooked the multifaceted functions and complexities of street spaces, particularly the importance of pedestrian areas and slow traffic zones that serve as hubs for various services and activities. With the advent of Internet of Things (IoT) technology, the conceptualization of urban street spaces has progressively shifted from a "car-oriented” to a "people-oriented” approach. This new perspective emphasizes the scientific allocation of street space resources to balance motor vehicle traffic needs while prioritizing pedestrian comfort. The ultimate goal is to create public spaces that encourage social interaction and prolonged stays by citizens [3,4]. In the context of urban development aimed at green, low-carbon, and humanized transformation of street spaces, the integration of new technologies like IoT and Artificial Intelligence (AI) is becoming increasingly significant. IoT technology facilitates the real-time collection and monitoring of street spatial data. Using sensors and monitoring equipment, it gathers various types of data, including traffic flow and environmental quality, to provide planners with precise information. AI technology complements this by enabling intelligent analysis and processing of street spaces through methods such as image recognition and semantic segmentation. This capability allows for the visual perception and evaluation of street view images' greenery. A notable application in this field is the Green Looking Ratio (GLR), which quantifies the three-dimensional green volume of street spaces based on people's psychological perceptions of their environment. The GLR visually represents these green volumes through quantitative indicators, enhancing the assessment of urban street environments. The rapid advancements in technologies like IoT-based Internet street views and deep neural network-based image recognition have significantly expanded the GLR's application scope, providing more comprehensive and nuanced evaluations of urban green spaces.

With the rapid advancement of urbanization, urban street spaces face significant ecological and aesthetic challenges. Traditional street planning methods often overlook environmental sustainability and resident satisfaction. In recent years, although research has begun focusing on using new technologies to optimize urban environments, effective integration of AI, deep learning, and IoT technologies into urban street planning remains an underexplored area. Furthermore, existing literature lacks systematic studies on the relationship between GLR in street space greening and resident satisfaction. Therefore, this study aims to fill this study gap by constructing deep learning models and integrating IoT technology to achieve efficient greening of street spaces and enhance resident satisfaction. The study is motivated by recognizing the limitations of current urban street planning methods and exploring the potential of new technologies. Particularly, the advantages of IoT technology in real-time data acquisition and processing, coupled with the robust capabilities of deep learning in image processing and pattern recognition, offer new possibilities for optimizing urban street spaces. This study seeks to provide a new perspective and methodology for urban street space planning, aiming to create more humanized and environmentally friendly urban living environments.

Building on the aforementioned context, this study begins by highlighting the significance of street spaces within cities and delves into classifying and grading GLR for these areas. It examines the workflow of the Fully Convolutional Network (FCN). This study innovatively proposes a GLR evaluation framework that integrates IoT and AI, utilizing FCN's image analysis techniques. The GLR calculation spans beyond individual green pixels to encompass five elements: trees, grasslands, plants, flowers, and terrain, providing a more precise reflection of street greenery. Leveraging Baidu Street View images, the study practically evaluates GLR in the historical urban area of T City, offering robust decision-making support for urban planners to enhance visual greenery quality. This study pioneers GLR evaluation in urban street spaces, addressing gaps in research and holding significant theoretical and practical implications. It establishes GLR benchmarks tailored to street conditions, thereby improving urban street aesthetics and serving as a valuable reference for developing humane, green ecological street environments.

## 2 Literature review

The study on GLR originates from visual environmental science and environmental psychology. Initially focused on natural areas, it has gradually shifted to the evaluation of urban space construction, with a primary emphasis on urban green spaces. For instance, Enssle and Kabisch (2020) investigated the modes of access to urban green spaces for the elderly in Berlin. Their findings indicated that elderly individuals with close social networks utilized urban parks more frequently than those who were more socially isolated. Additionally, self-perceived good health was associated with more frequent park usage. They concluded that urban planning should consider the city as a comprehensive social ecosystem, where the design and planning of urban green spaces aim to provide ecosystem services and foster social networks to enhance social and environmental justice [5]. Reyes-Riveros et al. (2021) conducted a systematic bibliographic review to explore the relationship between specific characteristics of green spaces and components of human well-being. Their analysis revealed that the quantity of green spaces, along with their vegetation coverage and area, significantly improved various aspects of human well-being, particularly health. Moreover, the biodiversity and naturalness of green spaces were found to enhance well-being by promoting health and fostering good social relations [6]. Semeraro et al. (2021) highlighted both the benefits and limitations of incorporating ecosystem services into green space design. They emphasized the dynamic feedback between ecological processes and functions that support ecosystem services and the urban environmental pressures that could potentially harm human well-being. Their study also discussed key considerations for planning and designing urban ecosystem services [7]. While these studies address various aspects of urban green spaces, they do not specifically focus on the GLR of urban street spaces. Therefore, this study begins with an analysis of the GLR in urban street spaces and examines the planning and design strategies for enhancing the green appearance of street environments.

In the context of rapid information technology advancement, the importance of IoT and spatiotemporal big data in urban spatial planning and understanding continues to grow. Recent studies, such as that by Salazar-Miranda et al. (2023), proposed new frameworks for real-time monitoring of street activities using machine learning and computer vision technologies [8]. This aligns with the objectives of this study, which utilizes IoT technology and deep learning neural networks for urban street space planning and design. By collecting and analyzing images from mobile vehicles, Study 1 demonstrates significant differences among different streets in supporting pedestrian activities, providing practical data for understanding and improving street designs. Similarly, using IoT technology to collect street view images and applying the FCN-8s framework for semantic segmentation, this study evaluates and enhances street greenery satisfaction. The alignment in methods and goals underscores the extensive application prospects of machine learning and computer vision in street planning and urban design. Additionally, Fan et al. (2023) explored urban life understanding through urban appearance studies [9]. Analyzing 27 million street view images from 80 counties in the US using computer vision models revealed correlations between urban visual features (such as street furniture, sidewalks, building facades, and vegetation) and socioeconomic characteristics, resonating with this study's investigation into the correlation between historical urban area street greenery ratios and satisfaction. The concept of urban visual intelligence highlighted in this study emphasizes the importance of visual data in urban studies, providing theoretical support for evaluating and improving urban greenery through street view image analysis, positioning this study within broader discussions on urban visual intelligence and big data applications. Moreover, Li et al. (2023) focused on detecting abandoned buildings in cities, showcasing the application of deep learning image segmentation methods in urban management [10]. While their primary focus was on abandoned buildings, their approach of using street view images for large-scale, fine-grained data collection and analysis aligns closely with the technical path of this study. By evaluating street greenery conditions through IoT technology and deep learning models to enhance urban street ecological environments, both studies share common ground in utilizing emerging data sources for refined urban management. This underscores the broad application prospects of deep learning and image segmentation technologies in urban planning, providing robust support for the feasibility and practicality of this study's research objectives. This study aims, against the backdrop of IoT, to explore and implement AI and deep neural network-based urban street space greening planning and design. Experimental data collection and analysis of street view images from the historical urban area of T City are conducted, employing the FCN-8s framework for semantic segmentation to assess GLR and investigate resident satisfaction. This process not only relies on IoT technology's efficient data collection capabilities but also showcases the potential of deep learning in processing and interpreting complex urban data. In contrast to existing research, the proposed approach not only focuses on using image data to identify and analyze urban issues but also integrates IoT and AI technologies to optimize urban street ecological environments and aesthetic layouts. For example, this study fully considers residents' actual perception and satisfaction regarding street greenery, supporting urban planning improvements and enhancing residential quality with empirical data. In addition, aligned with Salazar-Miranda et al.'s research, the experiment utilized IoT devices to collect real-time data and processed and analyzed the data using deep learning models. This approach not only enhances data processing efficiency but also improves the accuracy and practicality of the research. Such application not only responds to current trends in IoT and big data applications in urban planning but also explores the practical potential of these technologies in enhancing urban ecology and resident satisfaction.

In summary, existing research exhibits gaps and challenges in several areas: firstly, a lack of quantitative studies on specific GLR and its visual impacts; secondly, a predominance of theoretical frameworks and conceptual discussions with insufficient empirical data support; thirdly, limited research scope and application coverage, failing to comprehensively address various aspects of urban greening and ecological environments. These shortcomings indicate the need for an approach that integrates IoT and AI technologies to achieve comprehensive and refined assessment and optimization of urban greening and street spaces. This study innovates and contributes in several ways: firstly, efficiently utilizing IoT technology to collect street view images from the historical urban area of T City and performing semantic segmentation using the FCN-8s framework to quantify the GLR of street spaces. Secondly, by investigating resident satisfaction with GLR and combining empirical data, it reveals a significant positive correlation between GLR and resident satisfaction. This process not only enhances data processing efficiency and accuracy but also strengthens the practicality and application value of the study findings. Additionally, the study proposes specific recommendations for improving street greenery, such as adding planting ponds and boxes to "blind spots” and "dead corners” to enhance green layout and improve resident satisfaction and quality of life. By integrating IoT technology with deep learning neural networks, this study not only aligns with current trends in information technology applications in urban planning but also expands the practical potential of these technologies in enhancing urban ecology and resident satisfaction. The innovation of this study lies in its comprehensive application of multiple cutting-edge technologies, proposing feasible improvement solutions and providing important theoretical and empirical references for future urban greening and ecological planning.

## 3 Construction of GLR analysis model for street space

### 3.1 The classification of street space

Streets constitute vital public spaces within cities, playing a pivotal role in urban development. Understanding a city necessitates an exploration of its thoroughfares [11], analogous to essential vascular networks coursing through urban landscapes. The vitality of a city is intrinsically linked to the vibrancy of its streets [12], which serve as critical connectors between urban spaces. Moreover, streets serve as the primary public realm, facilitating communal interaction and fostering urban identity.

A clear classification of street types is fundamental to the effective design of street spaces. The greening layout and plant selection vary significantly across different street types, leading to diverse GLR in street spaces [13]. Therefore, a rational design approach must consider the street's traffic function, the nature of the land, the functional uses of the adjacent buildings, and the activities occurring along the street. Based on road traffic planning and the specific characteristics of street sections, street spaces can be categorized into four types: traffic-type streets, landscape-style streets, historical streets, and life leisure streets [[14], [15], [16]]. Traffic-type streets are characterized by non-open interfaces, wide roads, heavy traffic flow, and high-speed traffic, such as urban trunk roads, express lanes, and some high-traffic secondary urban roads. Landscape-style streets are generally quiet and stable, featuring prominent landscape and historical elements. These streets host a wide variety of activities, with public leisure facilities commonly located along them, and the types and timing of activities being relatively regular. Historical streets are distinguished by their unique landscapes that reflect the city's history and culture. These streets, alleys, and cultural trails maintain the scale and function of traditional street spaces and possess a strong cultural atmosphere. Life leisure streets are primarily found in residential areas, cultural and educational zones, service business areas, and public service sectors. They typically consist of secondary trunk roads and branch roads, characterized by complex traffic conditions, low traffic speeds, and high traffic volumes.

### 3.2 GLR analysis of street space

In urban settings, the architectural interface and GLR are pivotal factors influencing human visual perception. While the architectural interface of streets is typically fixed and challenging to alter, the GLR presents a primary opportunity for enhancing the visual experience of streets [17,18]. Coined by Japanese scholar Yoji AOKI, GLR quantifies the proportion of green plant coverage within the human visual field, offering a measurable metric [19]. Beyond aesthetic considerations, GLR profoundly impacts both physical and mental health. Studies indicate that regions with higher life expectancy often maintain GLR exceeding 15 %, whereas areas with GLR below 5 % face more than double the risk of respiratory-related mortality compared to those with GLR surpassing 25 %. A GLR exceeding 25 % is considered optimal for creating a visually pleasing green environment [20,21]. GLR classifications typically range across five levels (see Table 1) [22], providing a structured framework for evaluating and implementing green space strategies in urban planning and design (see Table 2).

**Table 1:** Grading of GLR satisfaction in street space.

GLR	Grade description
<5 %	Can hardly feel the environmental green quantity
5%–15 %	Poor perception of environmental green quantity
15%–25 %	The general perception of environmental green quantity
25%–35 %	Relatively strong perception of environmental green quantity
>35 %	Strong perception of environmental green quantity

**Table 2:** The role of IoT in street space planning.

Role	Description	Example
Real-time data collection and monitoring	Collect real-time data on street space, such as traffic flow, environmental quality, and energy use.	Monitor traffic flow through sensors, optimize traffic signal timing, and improve traffic efficiency.
Intelligent traffic management	Apply IoT technology to realize intelligent traffic management system. Optimize traffic lights, predict and manage traffic congestion,and improve street traffic through real-time data collection and analysis.	Reduce traffic congestion and improve road efficiency with real-time traffic data and smart signal systems.
Environmental monitoring and control	Use IoT technology to monitor the environment of street space, such as air quality, noise level, and temperature, understand environmental conditions, and formulate environmental protection and improvement measures.	The air quality and noise level are monitored by sensors to improve the environmental quality of streets and the life quality of residents.
Public safety and emergency management	Conduct public safety monitoring and emergency management. Through the deployment of sensors and monitoring equipment, the safety status of street space is monitored in real time, and early warning and response are carried out.	Through video surveillance and sensor monitoring, timely alarm and emergency measures are taken to ensure public safety.

Table 1 delineates different levels of GLR, reflecting varying degrees of people's perception of greenery in the environment. When GLR is less than 5 %, the perceived amount of greenery is negligible, indicating minimal green coverage that fails to provide a distinct green visual experience. GLR between 5 % and 15 % result in poor perception of greenery, with green effects being relatively insignificant. GLR from 15 % to 25 % lead to a moderate perception of greenery, where green effects become discernible. GLRs between 25 % and 35 % enhance perception significantly, offering a comfortable green visual enjoyment. GLR exceeding 35 % intensify green perception strongly, indicative of high green coverage that fosters a rich green environment and favorable visual experience.

GLR expands the research scope beyond traditional two-dimensional green spaces to encompass three-dimensional urban greening levels, considering people's psychological responses. It facilitates quantitative analysis of visual landscapes, introducing new metrics for evaluating urban street space greening [23,24]. The GLR value is determined by calculating the proportion of visually perceived green content within the total image pixels [25], encompassing trees, grass, plants, flowers, and natural features as visual green elements. This comprehensive approach covers the entirety of greening structures within the image, providing a standardized calculation as shown in Equation (1):

(1) $$P=\frac{G_{n}}{S_{n}}=\frac{\sum_{i=1}^{4}\sum_{j=1}^{3}\left(A_{t\_ij}+A_{l\_ij}+A_{p\_ij}+A_{f\_ij}+A_{m\_ij}\right)}{\sum_{i=1}^{4}\sum_{j=1}^{3}\left(A_{z\_ij}\right)}$$

in Equation (1), $G_{n}$ is the sum of the total pixels of all plant elements identified in the n-th street view image. $S_{n}$ is the total pixel of the n-th streetscape image. *A* is the image recognition area. *t*, *l*, *p*, *f*, *m* are trees, grass, plants, flowers and mountains, respectively. $A_{z}$ is the total element.

### 3.3 Application of IoT technology in street space planning

IoT constitutes a technology ecosystem facilitating data exchange and communication among interconnected physical devices, sensors, software, and networks. This interconnectedness enables the integration of various devices and objects, enabling intelligent data collection, analysis, and application [26]. In the realm of street space planning, IoT holds significant promise, offering numerous potential roles and advantages for urban planning and street design. By connecting sensors, embedded systems, and the Internet, IoT facilitates the perception, data collection, and interconnection of the physical environment. These devices can monitor environmental changes, gather location data, collect traffic statistics, and assess air quality. Through the systematic collection, storage, and analysis of such data, IoT equips city planners with real-time, accurate insights to inform more informed decision-making processes.

**Fig. 1:**
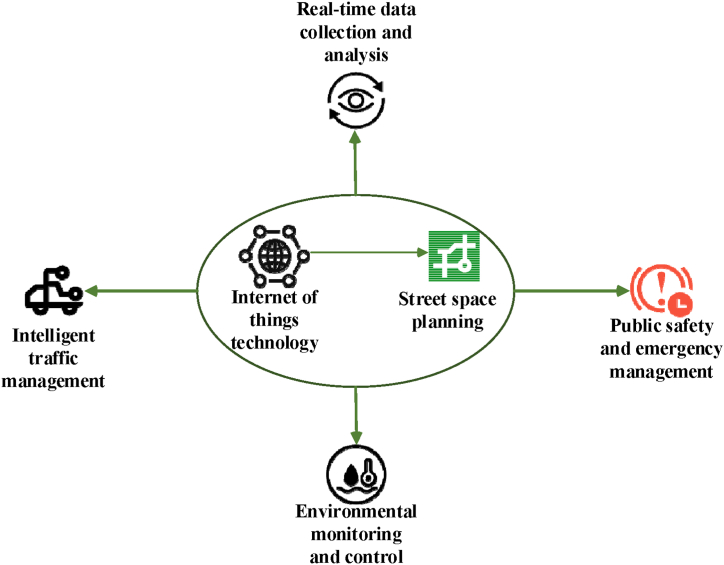
The role of IoT in street space planning.

The integration of IoT technology in urban planning embodies the concept of smart cities, fostering more efficient, sustainable, and livable urban development. By incorporating IoT across various facets of urban planning—such as land use, transportation, environmental management, and public facilities—cities can leverage real-time data to better understand urban dynamics and resident needs, thereby formulating more adaptive planning strategies. Fig. 1 illustrates the potential roles of IoT in optimizing street space planning and enhancing urban environments.

In Fig. 1, the IoT enables perception, data collection, and interconnection of the physical world by connecting sensors, embedded systems, and the Internet. These technologies can sense environmental changes, obtain location information, collect traffic data, and measure air quality. Through the collection, storage, and analysis of data, IoT provides city planners with more real-time and accurate information to help them make wiser decisions. Additionally, the application of IoT technology contributes to realizing the concept of smart cities. By integrating IoT into urban planning, cities can achieve more efficient, sustainable, and humane development. Details are given in Table 2.

Table 2 illustrates the multifaceted roles of IoT technology in street space planning, offering capabilities for real-time data collection and monitoring. Sensors, for instance, gather real-time data on street space such as traffic flow, environmental quality, and energy usage. This enables monitoring of traffic flow through optimized traffic signal timing, enhancing overall traffic efficiency. IoT applications also enhance smart traffic management by collecting and analyzing real-time data to optimize traffic signals, predict and manage congestion, thereby reducing traffic jams and improving road efficiency. Environmental monitoring and control represent another vital application of IoT technology, facilitating the monitoring of street space environments including air quality, noise levels, and temperature. This data informs environmental assessments and supports the formulation of environmental protection and improvement measures. For instance, monitoring air quality and noise levels via sensors can improve street environment quality and residents' quality of life. Public safety and emergency management are also critical roles of IoT technology, with sensors and monitoring devices deployed for real-time monitoring of street space safety conditions, enabling timely alerts and responses. For example, video surveillance and sensor monitoring can promptly issue alerts and implement emergency measures to ensure public safety.

In summary, IoT technology holds substantial potential for application in street space planning. By leveraging IoT technology, urban planners can access and analyze critical data such as street space utilization, traffic flow, and environmental quality. This facilitates the development of more scientifically grounded and effective street planning and design strategies aimed at creating greener, sustainable, and more human-centered urban street environments.

The image data utilized in this study is primarily captured through IoT devices, providing a street-level perspective. However, it is crucial to acknowledge potential limitations due to fixed device positions and environmental constraints affecting image quality and perspective coverage. Firstly, the placement of IoT devices may restrict the visual range captured, thereby influencing image coverage and quality. Variances in device locations can lead to inadequate data coverage in certain areas, posing challenges for subsequent image segmentation and analysis processes. Secondly, image quality captured by devices may be affected by environmental conditions such as lighting and weather, impacting clarity and detail capture. These factors directly influence the accuracy of segmentation algorithms and the reliability of subsequent analyses. To address these challenges, it is recommended to employ multiple IoT devices positioned at different locations and angles during data collection to enhance data diversity and coverage. Furthermore, regular maintenance and calibration of devices are essential to ensure captured image quality meets required analytical standards. In conclusion, while IoT devices offer real-time street-level perspectives for this study, addressing and mitigating issues of image quality and perspective limitations are crucial to ensuring data accuracy and analysis reliability.

### 3.4 FCN-based semantic segmentation framework model

**Fig. 2:**
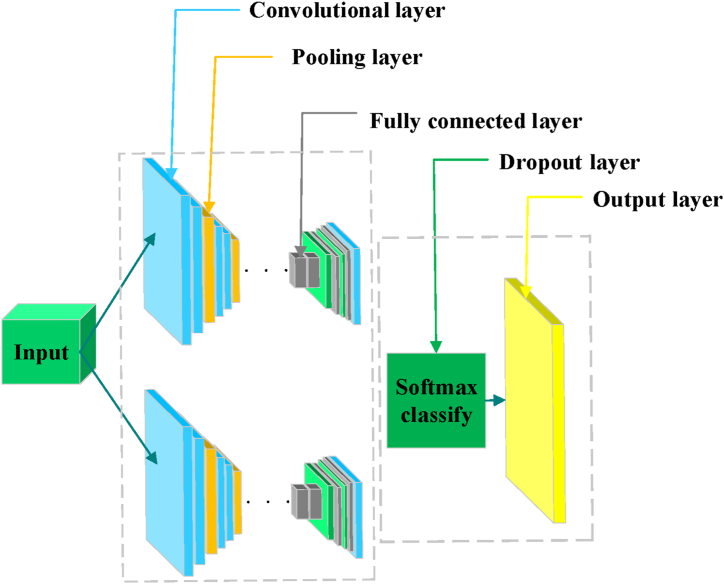
FCN workflow.

The Convolutional Neural Network (CNN) represents a powerful deep learning algorithm that has achieved significant advancements and is widely applied in tasks such as image recognition, classification, and object detection. One prominent application of CNN-based deep learning technology is Image Semantic Segmentation, which enables the assignment of semantic labels to individual pixels within an image, thereby facilitating semantic understanding [27]. In the context of urban street space analysis, commonly utilized semantic segmentation frameworks include FCN, Semantic Segmentation (SegNet), and Pyramid Scene Parsing Network (PSPNet). These frameworks utilize deep learning models trained on extensive public datasets to accurately and efficiently identify semantic elements within street view images, surpassing the capabilities of traditional manual recognition methods [28,29]. FCN, recognized as a milestone in image semantic segmentation technology, is employed in this study for semantic segmentation of street view images. Fig. 2 illustrates the workflow of FCN, showcasing its process in segmenting and labeling semantic elements within street view imagery [30].

Fig. 2 illustrates that the core principle of FCN is to replace the fully connected layers found in traditional CNN architectures with convolutional layers. This modification allows for the resolution of the image to decrease progressively through multiple convolutional operations. Subsequently, the network performs up-sampling through deconvolution, scaling the feature maps back to the original image size by factors of 32, 16, and 8. This process culminates in optimized output that maintains two significant advantages: enhanced operational efficiency and the capability to process images of variable sizes [31,32]. FCN-8s, a variant tailored for semantic segmentation tasks, extends this concept further. Like FCN, FCN-8s replaces traditional fully connected layers with convolutional layers, facilitating the model's ability to handle input images of arbitrary dimensions while producing semantic segmentation results of corresponding dimensions. The workflow of FCN-8s can be delineated into several critical steps [33].

1) Input Image: FCN-8s begins with an image input, which can be of any size and in color. This flexibility in handling images of varying dimensions is a fundamental characteristic of FCNs.
2) Feature Extraction: In FCN-8s, feature extraction leverages a pre-trained CNN to extract image features. This stage involves multiple convolutional and pooling layers that progressively reduce the size of the feature map while preserving important spatial information.
3) Multi-scale Feature Fusion: FCN-8s integrates multi-scale feature maps derived from different layers and depths of the network. These feature maps encapsulate semantic details at various scales. To fuse them effectively, FCN-8s employs strategies such as upsampling and skip connections. Upsampling enlarges low-resolution feature maps to match the input image size, while skip connections merge coarse low-level feature maps with refined high-level feature maps.
4) Semantic Segmentation Prediction: Using convolutional operations, FCN-8s combines the fused feature maps to generate segmented maps. Each pixel in the segmented map corresponds to a pixel in the input image and is assigned a semantic category, achieving the task of semantic segmentation.
5) Output of Segmentation Results: The resulting segmentation map from FCN-8s provides semantic categorization for every pixel in the input image. These segmented outputs are valuable for further processing, such as visualization, object detection, or other relevant applications.

The FCN-8s model offers two primary advantages. Firstly, it accommodates input images of varying sizes, enhancing its practical flexibility by eliminating the need for resizing images to a fixed dimension. Secondly, its fully convolutional structure enhances operational efficiency, reducing model complexity. This model has demonstrated effective recognition capabilities in challenging environments, including under-construction areas, rural regions, and urban streets of Wuhan City [34], making it well-suited for the present recognition task.

To mitigate neural network overfitting in street view image semantic segmentation tasks, this study employs several measures during model training. Initially, training utilizes the diverse ADE20K Scene Parsing Dataset, which includes a plethora of complex scenes to enhance model generalization. Additionally, regularization techniques such as L2 regularization penalize model complexity by constraining weight magnitudes, thereby averting overfitting. Furthermore, early stopping criteria halt training when validation performance plateaus, preventing the model from excessively fitting the training data. These strategies collectively ensure robust recognition performance and generalization to unseen data.

The deep learning FCN-8s model is trained using the ADE20K dataset for image semantic segmentation tasks. This dataset comprises 25,574 training images and 2000 validation images, encompassing diverse scenes like outdoor and indoor settings, urban streets, and natural environments, categorized into 151 semantic elements. Given the resource-intensive nature of dataset construction frameworks, this study leverages the FCN-8s framework developed by the High Performance Computing Laboratory of China University of Geosciences. The model achieves an 81.44 % pixel-wise accuracy on the training dataset and attains a 66.83 % accuracy on the test dataset in pixel-wise comparisons.

This study employs the classical deep learning framework FCN-8s for semantic segmentation of street view images. Within this framework, three main components are utilized: convolutional layers for feature extraction using convolution kernels, deconvolutional layers for upsampling to the original image dimensions, and skip connections to refine predictions by integrating low-level feature maps with high-level feature maps for more accurate predictions. In practical application, street view images are initially input into the CNN, where successive convolution and pooling operations abstract spatial information into high-level features. Subsequently, deconvolutional layers map these high-level features back to the original image size to predict the category for each pixel. Integrating low-level feature information enhances the model's prediction accuracy. Finally, the Softmax function computes the probability of each pixel belonging to the background or foreground, thereby evaluating the model's performance. During model training, the cross-entropy loss function is employed as the optimization objective, and stochastic gradient descent (SGD) is utilized for updating model parameters. The initial learning rate is set to 0.001, and it decreases by 10 % every 10,000 iterations throughout the 50,000 iterations of training. Additionally, momentum factor 0.9 and weight decay factor 0.0005 are applied to accelerate model convergence and prevent overfitting.

### 3.5 Experimental data collection

To ensure the accuracy and reliability of data collection, this study employs a multi-source data validation approach. Initially, road network data from OpenStreetMap (OSM) serves for the preliminary assessment of street space in the historical urban area of T City. Concurrently, real-time data on traffic flow and environmental quality collected through IoT technologies dynamically adjust the classification of street types. Additionally, street view images are systematically collected using random sampling methods from various locations and time points to ensure the representativeness and diversity of samples. This approach effectively mitigates biases from single data sources, enhancing the objectivity and precision of analysis results. The historical urban area of T City serves as the study area, where residential land occupies 33.8 % of the total area, and commercial and service land cover 20.2 %. Together, these two categories exceed half of the area, while park green space constitutes only 11.6 %, with no dedicated land for cultural facilities or public squares. This distribution underscores the area's primary function as residential, highlighting a significant need for green space. Moreover, there is a lack of cultural amenities, recreational areas, and public green spaces to serve the community's leisure needs.

**Fig. 3:**
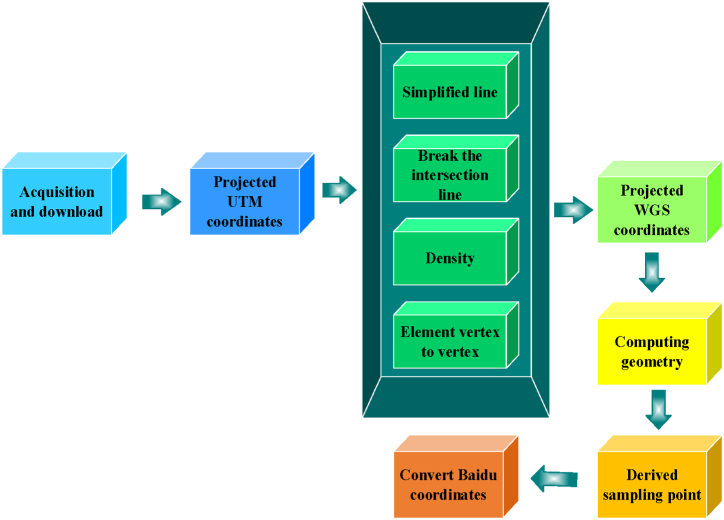
Collection and processing steps of road network data.

OSM serves as the primary source for road network data in T City's historical urban area. OSM is a freely editable and open-source mapping platform containing comprehensive data on highways, roads, railways, water systems, and buildings [35]. Its road network data's reliability has been validated by multiple researchers and is widely accepted across various scientific studies. Fig. 3 illustrates the steps involved in collecting and processing road network data [36], demonstrating its utility in urban research and planning contexts.

In Fig. 3, the process begins with logging into the OSM official website to manually select different regions and download road network data. The selected region's size is specified, and the data is imported and projected into the Universal Transverse Mercator Grid System (UTM) coordinates. Subsequently, the road network data undergoes simplification to approximate straight lines, reducing disorderly turning points, nodes, and polylines. Road elements are segmented at intersections, and the data are sampled and densified. Feature breakpoints are then used to extract line feature breakpoints, generating sample points that are projected into World Geodetic System (WGS) coordinates. Longitude and latitude coordinates of the sample points are computed, and an attribute table is exported. Finally, Excel is utilized to organize the sampling point data, and coordinate conversion software converts these points from geographic coordinates to Baidu coordinates. The dataset address is https://www.kaggle.com/datasets/hamzafar/look4me.

The integration of map data from OSM with real-time IoT technology enhances data collection efficiency. IoT sensors deployed in the historical urban area of T City facilitate the continuous real-time update and collection of road network data. These sensors monitor information such as traffic conditions, road usage, and traffic density, transmitting the data to servers for processing.

In street view image acquisition, IoT technology integrates with Baidu panoramic static images as the primary data source. Supported by IoT devices and sensors, real-time street space image data is acquired. IoT technology allows for flexible selection of image acquisition parameters, including image size, latitude and longitude coordinates, viewing angle, and direction, simulating the human eye's field of view in street spaces. This simulation aims to capture street view images of the historical urban street space. The crawling image pixels are set at 1024 * 512, with each sampling point collecting six street view images spanning a horizontal angle of 60°: 0°, 60°, 120°, 180°, 240°, and 300°. The horizontal field of view is 60°, with a vertical angle of 0°, resembling a binocular head-up display. This setup replicates the visual perception of urban street spaces under human eye perspective, facilitating analysis of street space composition and the GLR proportion.

To enhance the universality and reliability of experimental results, the survey expanded the selection range and quantity of sampling points. In addition to the existing framework, the experiment added 30 additional sampling points within the historical urban area of T City. These new points ensure coverage across different types of streets—such as traffic-oriented, landscape-oriented, historical, and leisure streets—as well as varied residential and commercial areas. This approach comprehensively reflects the current status of street space greening throughout the historical urban area and its impact on resident satisfaction. Furthermore, the image data collection not only increased the number of images per sampling point from 6 to 12, capturing views from multiple angles, but also optimized the timing of image acquisition—morning, noon, and evening—to assess the visual effects of street greening across different time periods. This enhancement aims to more accurately evaluate the impact of GLR on residents' daily living experiences. Moreover, random sampling techniques are introduced to select specific locations for image acquisition, enhancing sample randomness and representativeness. This method ensures that selected sampling points authentically and fairly represent the diversity and complexity of the entire study area. To validate the representativeness of sampling points and the effectiveness of experimental design, statistical analysis is employed. By calculating the geographical distribution uniformity of sampling points and their alignment with proportions of different street types, the experiment confirmed that the distribution of sampling points closely aligns with the actual conditions in the historical urban area, demonstrating high representativeness. Finally, thorough data cleaning and preprocessing are conducted on all collected image data to ensure data quality and the accuracy of experimental results.

To mitigate potential biases introduced by survey methods, a more rigorous and diversified approach is applied. Firstly, data collection involves not only online surveys but also on-site surveys to obtain more direct and genuine feedback, thereby reducing potential inconsistencies or biases in online responses. Additionally, to comprehensively understand respondents' perspectives on street space greening, open-ended questions are included in the questionnaire alongside a five-level satisfaction rating scale. This approach allows respondents to freely express their opinions and suggestions, aiming to capture nuanced viewpoints and enhance the richness and diversity of data. Concurrently, the sample size is expanded to cover more regions and demographics, ensuring the results are more representative and applicable. Furthermore, addressing the lack of control over external variables that can influence GLR or satisfaction scores in the research design, additional control variables are incorporated into the data analysis process. Based on relevant literature and research, factors potentially affecting GLR or satisfaction scores—such as street width, building height, and pedestrian traffic—are selected. Integrating these control variables into statistical models not only further tests hypotheses but also enhances the rigor and credibility of the study. This inclusion of external variables enables a more precise assessment of the true impact of street space greening on resident satisfaction, mitigating conclusion biases caused by external interference.

GLR satisfaction is assessed via a questionnaire survey structured into two parts. The first part gathers respondents' basic personal information, including their residential or activity scope, gender, and age. The second part employs a five-level satisfaction scale to evaluate the visual greening experience of street view images. The scale ranges from “very poor” (−2) to “very good” (2), allowing respondents to rate their subjective satisfaction with the displayed street view images. These images are selected from four categories: traffic-oriented, landscape-oriented, historical streets, and leisure streets, totaling 48 representative streetscape images (12 per category). The survey is conducted online via the Questionnaire Star platform, distributed through WeChat and Weibo to citizens residing or active in the historical urban area of T City. A total of 300 questionnaires were distributed, and after collection, statistical screening was performed on both the survey respondents and image scores. Ultimately, 265 valid questionnaires were obtained, yielding an effective response rate of 88.3 %.

## 4 Analysis of experimental results

### 4.1 Street space GLR distribution and GLR satisfaction results

**Fig. 4:**
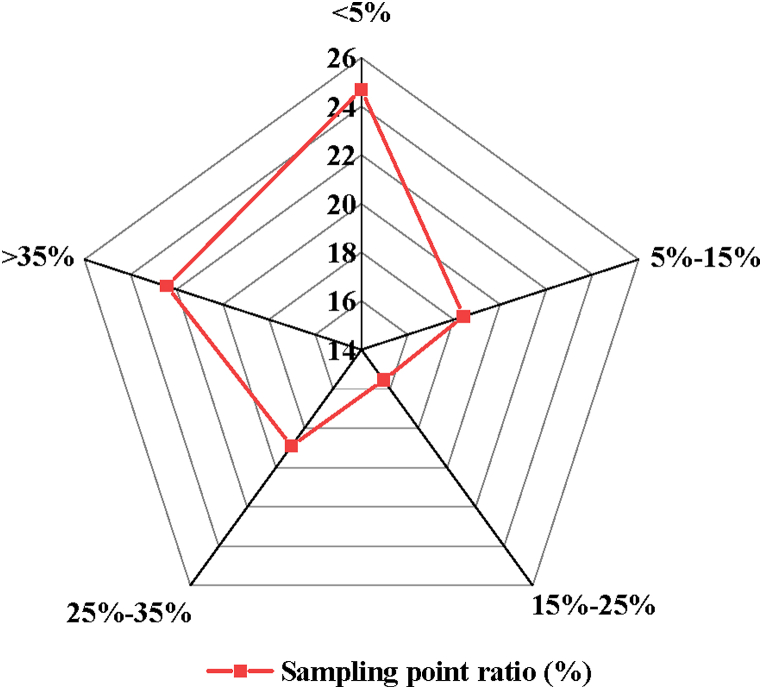
GLR distribution of street space in the historical urban area of T city.

According to the division of the five levels of GLR, the GLR distribution results of the street space in the historical urban area of T City can be obtained, as shown in Fig. 4.

Fig. 4 illustrates the distribution of GLR in the street spaces of T City's historical urban area. The data reveals that 24.69 % of sampling points have GLR values below 5 %, while 43.11 % fall within the range of 5 %–15 %. Only 15.56 % of sampling points have GLR values between 15 % and 25 %, whereas 33.07 % of sampling points exhibit GLR values exceeding 25 %, with 22.43 % of these points having GLR values surpassing 35 %. This indicates that the overall quality of street space greening in T City's historical urban area is relatively low, suggesting significant room for improvement.

**Fig. 5:**
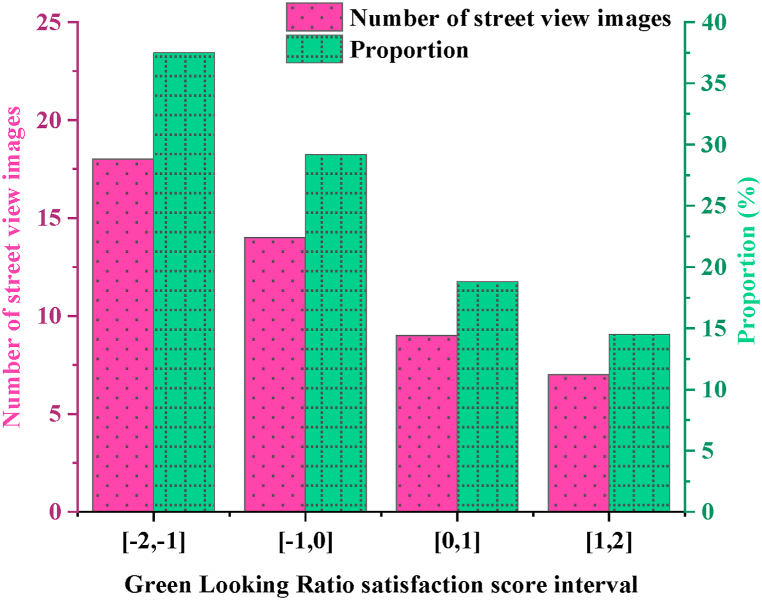
GLR satisfaction score of street space in the historical urban area of T city.

The GLR satisfaction score of 48 typical street view images selected through the network questionnaire survey is counted. Fig. 5 is the result.

Fig. 5 presents the GLR satisfaction scores obtained from an online questionnaire survey of 48 typical street view images. The results indicate that 18 street view images have an average GLR satisfaction score in negative values, with the majority falling in the [−2,-1] range, accounting for 37.5 %. Images with scores in the [−1,0] and [0,1] ranges represent 29.2 % and 18.8 %, respectively, while those in the [1,2] range are the least common, comprising only 14.5 %. This reflects a generally low satisfaction with the greening of street views, highlighting the need for improvement in GLR for street spaces in T City's historical urban area.

### 4.2 Comparative analysis of GLR and satisfaction

**Fig. 6:**
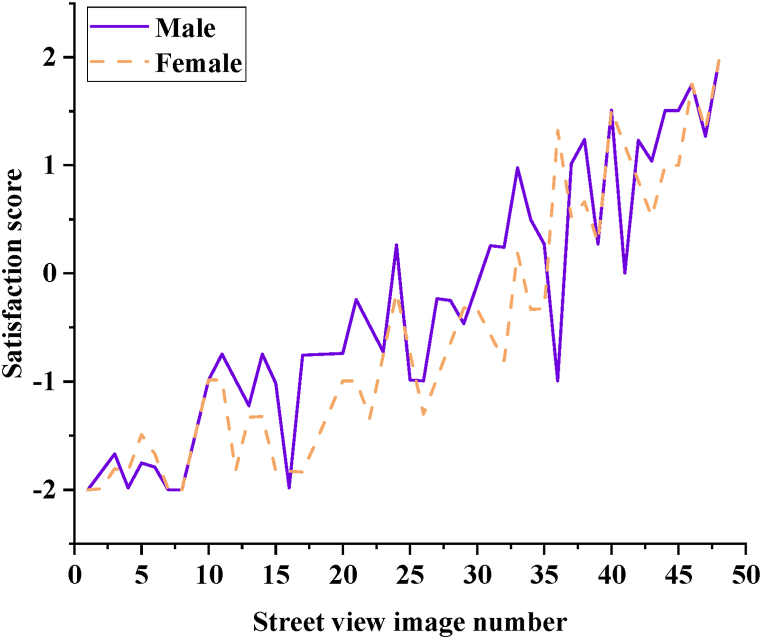
Distribution map of gender preference for satisfaction with street space GLR.

In the questionnaire survey, there are 120 men, accounting for 45.28 %, and 145 women, accounting for 54.72 %. The satisfaction of different genders with the GLR of street space in the historical urban area of T City is compared, as shown in Fig. 6.

In Fig. 6, both males and females show a similar increasing trend in GLR satisfaction scores, with males generally exhibiting higher scores than females, indicating slightly lower expectations for green visual environments in street spaces among males compared to females. Both genders consistently rate street view images as either “very good” or “very poor.” Specifically, images No. 40, 46, and 48 receive high satisfaction ratings from both groups. The data reveals fluctuating trends in satisfaction scores as the number of street view images increases. For instance, images with higher counts, such as 10, 11, and 13, show significant improvements in satisfaction scores. However, overall satisfaction remains relatively low, suggesting a need for further optimization in street greening and landscape design to enhance resident satisfaction. Street view images No. 1, 7, and 8 receive a score of −2, indicating very poor satisfaction levels, underscoring the need for improvement in GLR in T City's historical urban area. Gender does not significantly influence satisfaction with GLR in the historical urban area of T City, as both males and females similarly rate images No. 1, 7, and 8 poorly. However, there is a gender difference in GLR satisfaction for image No. 17, where females generally perceive the street view and green environment as poorer compared to males.

**Fig. 7:**
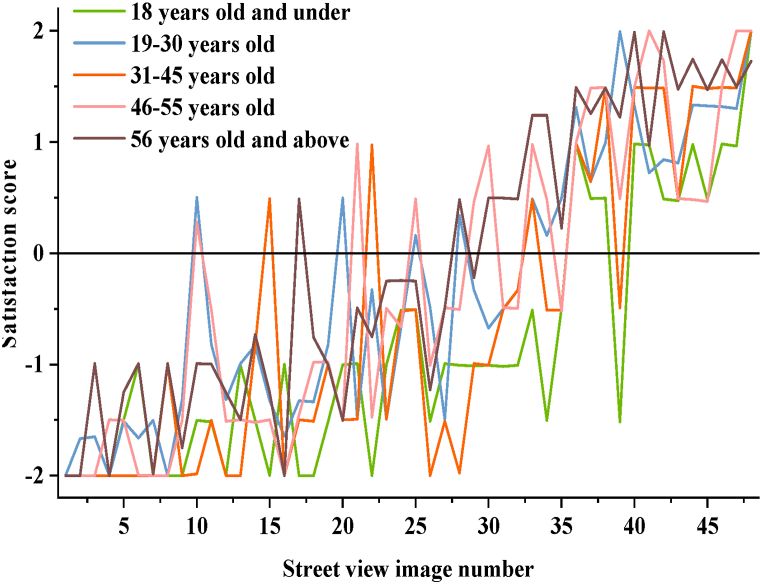
Distribution map of age preference for satisfaction with street space GLR.

Regarding age distribution, respondents are categorized as follows: 12.08 % aged 18 and below, 17.36 % aged 19–30, 29.81 % aged 31–45, 21.51 % aged 46–55, and 19.24 % aged 56 and above. Fig. 7 illustrates the distribution of satisfaction preferences across different age groups towards GLR in street spaces.

Based on Fig. 7, the data indicates that residents aged 56 and above, as well as those aged 18 and below, 19–30 years, 31–45 years, and 46–55 years, generally express lower satisfaction scores for street views, particularly among older adults and adolescents. This suggests significant differences in needs and preferences among different age groups, highlighting the necessity to consider these variations in street space planning to enhance overall resident satisfaction. The satisfaction scores for GLR across different age groups show similar trends, mirroring the patterns observed by gender. Citizens aged 18 and under exhibit the highest satisfaction rating for street view image No. 48, which is also the most satisfying street space for the majority of citizens. Street view images No. 47, 48, 41, and 42 receive the highest ratings from citizens aged 46 and above. Furthermore, citizens aged 18 and under and those aged 31–45 have the highest number of street view images rated as “very poor,” with 12 and 14 images, respectively. In contrast, other age groups rate approximately 5 images as “very poor,” indicating that younger age groups (18 and under) and those aged 31–45 have significantly higher expectations and usage frequency for green spaces compared to other age demographics.

**Fig. 8:**
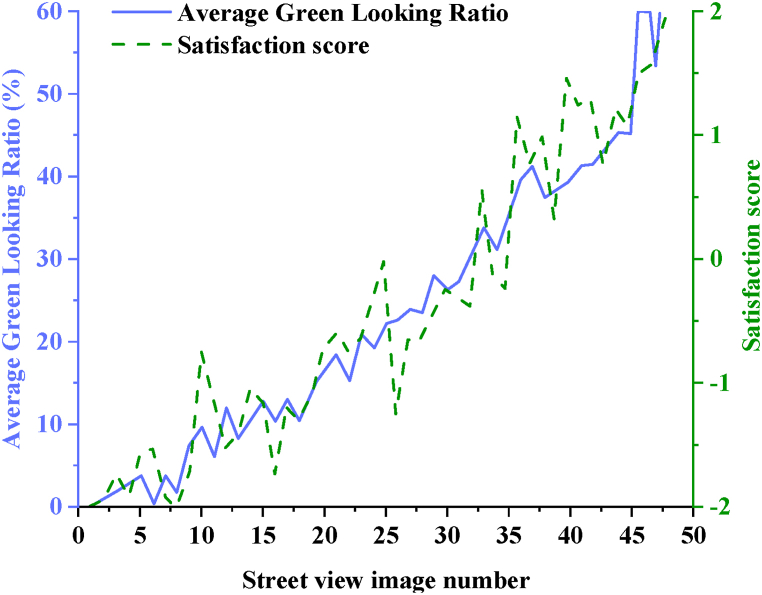
Comparison between street space GLR of the historical urban area of T city and satisfaction score.

Fig. 8 compares the average GLR of street view images used in the questionnaire with their corresponding satisfaction scores.

Based on Fig. 8, it is observed that the overall trend of satisfaction scores for street view images aligns closely with the average GLR. As the proportion of green visual elements increases, there is a corresponding improvement in satisfaction scores. However, even in cases where there is a high proportion of green visual elements, satisfaction scores may still be relatively low. This indicates that simply increasing the proportion of green visual elements alone may not fully meet residents' expectations, necessitating consideration of other factors such as landscape design and facilities configuration. To further explore the relationship between citizens' satisfaction scores for GLR across 48 typical street view images and the average GLR of these images, statistical analysis is conducted using SPSS 26.0 software. The analysis reveals a statistically significant correlation (p < 0.05) between the two variables, with a Pearson correlation coefficient of R^2^ = 0.919**. This indicates a strong positive correlation between the average GLR of street spaces in the historical urban area of T City and the satisfaction scores reported by citizens.

**Fig. 9:**
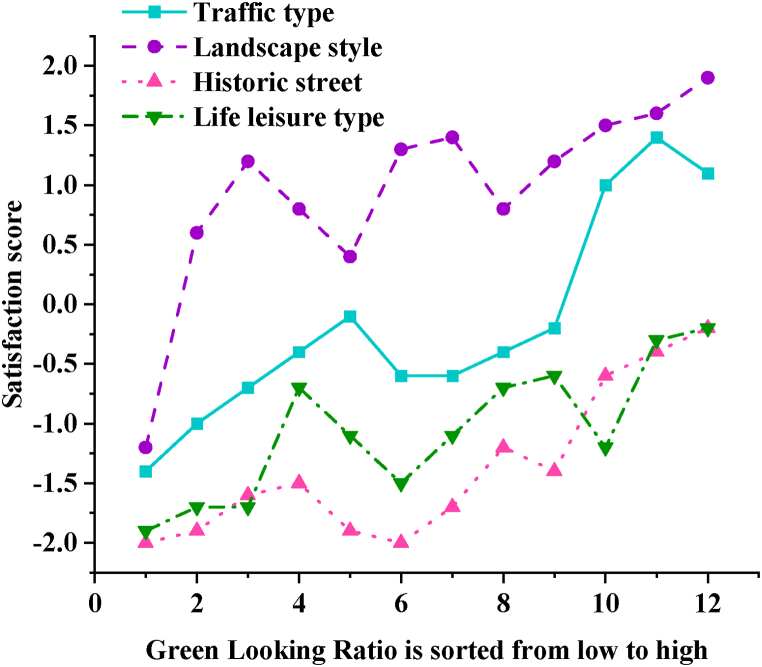
Comparison of GLR of different types of street space and satisfaction scores.

Fig. 9 categorizes and sorts the 12 typical street view images of each street space type in the questionnaire based on their GLR values from lowest to highest.

Based on Fig. 9, the results indicate that satisfaction scores for historical streets are generally lower, suggesting significant room for improvement in their greening and environmental enhancements. In contrast, landscape-style streets receive higher satisfaction scores, indicating that their greening effects and landscape designs are well-received by residents. The overall trend across the four types of street spaces shows a consistent increase in satisfaction scores with higher GLR values, but there are notable differences in the range of GLR satisfaction scores among citizens. Landscape-style streets exhibit the highest GLR satisfaction, with satisfaction scores for 11 street view images falling within the range of 0.5–1.5. This indicates relatively stable satisfaction levels overall, less influenced by GLR values. While satisfaction scores and GLR values for historical streets and life leisure streets are increasing, all street view images still receive negative satisfaction scores, indicating evaluations below "average”. This underscores the need for stronger greening efforts in street space construction.

### 4.3 GLR analysis of different types of street space

**Fig. 10:**
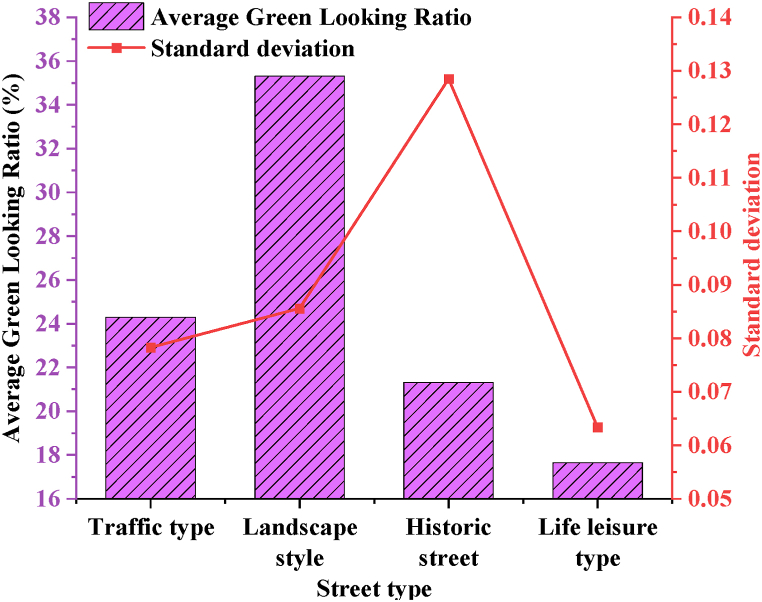
Average GLR and standard deviation of different types of streets.

The average GLR and standard deviation of different types of streets in the historical urban area of T city are counted. Fig. 10 displays the result.

Based on Fig. 10, landscape-style streets exhibit the highest average GLR at 35.31 %, reaching an overall "good” level of greening. This indicates a relatively uniform and stable greening effect across this type of street. In contrast, traffic-type streets and life leisure streets have lower average GLR values, suggesting substantial room for improvement in greening efforts for these street types. The life leisure street shows the lowest average GLR at 17.65 %, indicating a common overall satisfaction level. Despite being heavily utilized in citizens' daily lives, these streets exhibit lower levels of greening construction and a somewhat chaotic distribution. Historical streets display the highest standard deviation at 0.1285, indicating high volatility in their GLR values. This variability suggests that some historical streets feature high GLR values while others have very low values, reflecting inconsistent greening efforts across different areas. Some historical streets suffer from multiple issues, with minimal greening construction evident.

**Fig. 11:**
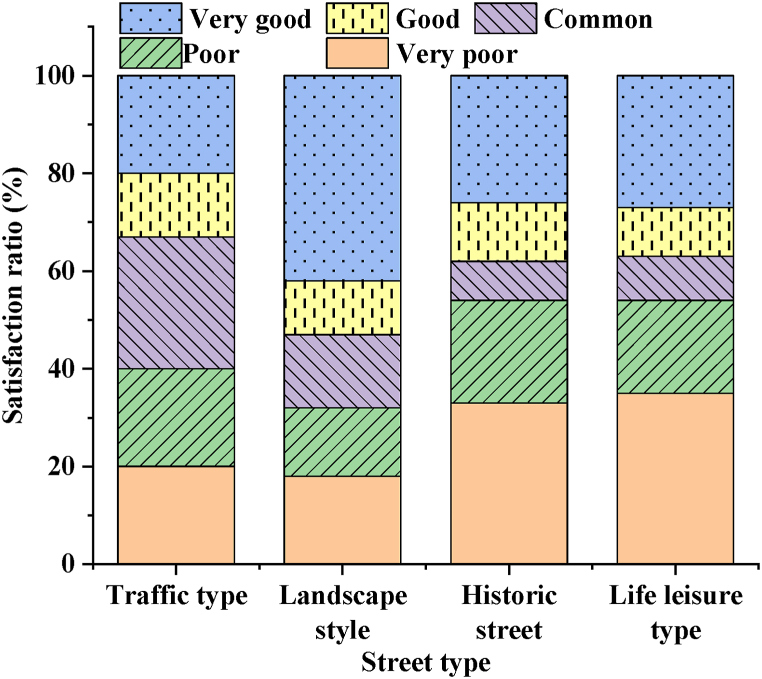
Comparison of GLR satisfaction of four types of streets.

For a comparison of GLR satisfaction across the four types of streets, refer to Fig. 11.

Fig. 11 illustrates that landscape-style streets exhibit the highest level of green-looking environment construction among the four types of street spaces. Regions rated as good and very good account for 53 %. The greening construction level of life leisure streets and historical streets is relatively low, with “poor” and “very poor” average GLR accounting for 63 % and 62 %, respectively. There is a significant gap compared to overall greening construction and the other two types of street spaces. The GLR of traffic streets is evenly distributed across all levels, indicating room for targeted local visual greening improvements. Landscape-style streets have the highest proportion in the “very good” rating category, at 42 %, while life leisure streets have the highest proportion in the “very poor” rating category, at 35 %. The result suggests that the design and greening effects of landscape-style streets are more popular among residents, while there is considerable room for improvement in enhancing resident satisfaction with life leisure streets.

**Fig. 12:**
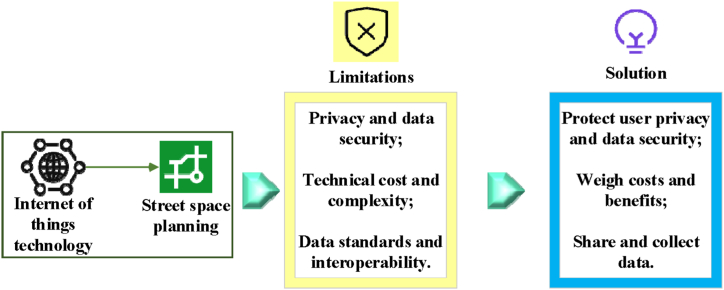
Limitations of IoT technology.

In addition, the experiment also reveals certain limitations of IoT technology in the application of street space planning, as depicted in Fig. 12.

Fig. 12 highlights some limitations of IoT technology in the application of street space planning. During data collection and transmission, IoT technology involves significant personal and sensitive information, making privacy protection and data security crucial considerations. When deploying IoT technology, it is essential to develop appropriate privacy protection measures and security mechanisms to prevent potential data leaks and misuse. The application of IoT technology requires investment in hardware devices, sensors, and network connectivity, which entails costs and complexities including equipment procurement, deployment, maintenance, and data management. Therefore, when applying IoT technology in street space planning, a balance between costs and benefits is necessary to ensure the feasibility and sustainability of the technology. Moreover, IoT technology involves data exchange and interoperability between different devices and systems. In street space planning, integrating multiple data sources and platforms for data sharing and integration may be necessary. Ensuring data consistency, standardization, and interoperability poses a challenge that needs to be addressed. The effective application and sustainable development of IoT technology require comprehensive consideration of these factors, along with the formulation of appropriate strategies and measures.

## 5 Discussion

This study integrates IoT technology and deep learning neural networks to explore the planning and design of urban street spaces, with a particular focus on the influence of GLR. Based on empirical data from a historical urban area, this study employs FCN for semantic segmentation and analyzes citizen satisfaction through questionnaire surveys to propose specific measures for improving street greenery. To provide a comprehensive understanding and validation of the study's findings, comparisons and discussions with related studies by other scholars in the field are incorporated. For instance, Chen et al. (2021) highlighted that urban green environments directly impact residents' mental health and life satisfaction [37], further supporting the conclusions of this study. Yang et al. (2023) conducted a detailed analysis of the relationship between street space GLR and resident satisfaction [38]. He et al. (2019) used a multivariate regression model to explore various factors affecting resident satisfaction, including socioeconomic indicators, environmental quality, and urban infrastructure [39]. Building on this approach, additional control variables such as neighborhood noise levels and pedestrian density were introduced in this study to examine their moderating effects on the relationship between GLR and resident satisfaction. In contrast, Bai et al. (2022) assessed urban green spaces using remote sensing imagery and GIS technology [40]. They primarily focused on macro-level distribution and area calculations of green spaces, statistically analyzing the correlation between urban green space coverage and residents' quality of life. However, their study did not delve into specific street greenery construction details and their impact on visual experiences for residents. In comparison, this experiment utilized IoT technology for real-time collection of street view images and employed the FCN-8s framework for image semantic segmentation, achieving micro-level calculations of green ratios. This approach not only enhanced data accuracy and real-time capability but also provided more intuitive insights into greenery in citizens' daily living environments. Additionally, Harris et al. (2021) proposed the application of smart sensors in urban management, monitoring traffic flow, air quality, and other information through sensor networks [41]. They emphasized the advantages of IoT technology in real-time data updates and multi-source data integration but did not apply it to the analysis of urban greenery. This experiment further expands the application scope of IoT technology by integrating street view image acquisition and citizen satisfaction surveys, systematically analyzing GLR of different types of streets and their impact on resident satisfaction. Experimental results indicated that landscape-style streets had the highest GLR and satisfaction, while life leisure streets and historical streets had lower satisfaction rates, providing a basis for future green planning. Furthermore, Timilsina et al. (2020) discussed deep learning-based methods for urban greenery recognition, using CNN to classify urban images [42]. Their experiment contributes to the technological foundation of automated greenery assessment, complementing the methodological advancements highlighted in this study. It contributes to the field by integrating IoT technology and deep learning for detailed analysis of street space greenery, providing insights into improving urban planning and enhancing resident satisfaction. Future research could explore further applications of advanced technologies in urban greenery assessment and planning, considering broader environmental and societal impacts. Although they have achieved certain achievement in green space recognition, their models primarily target macro urban areas and lack attention to specific street space details. This study, using the FCN-8s framework, not only achieves fine-grained processing of streetscape images but also analyzes citizens' subjective perceptions of different GLR through a questionnaire survey. The results show a significant positive correlation between GLR and citizen satisfaction, especially on landscape-style streets, where an increase in GLR significantly enhances visual satisfaction. This finding confirms the importance of greenery in improving urban environmental quality and provides a scientific basis for specific street greening planning. In summary, based on previous research, this study has several innovations and advantages: Firstly, by integrating IoT technology with the FCN-8s framework, it achieves a detailed analysis of the greening proportions of different types of streets, providing data support for urban street space planning and design. Secondly, by combining streetscape images with citizen satisfaction surveys, it systematically reveals the relationship between GLR and citizen satisfaction, providing a scientific basis for urban greening policy formulation. Furthermore, the study suggests adding planting pools and planting boxes in "blind spots” and "dead ends” to improve areas lacking greenery, offering highly practical recommendations. Through comparison and analysis with existing literature, the study not only confirms that the GLR in street spaces has a significant positive impact on residents' satisfaction but also reveals the complexity and multidimensionality of this relationship. This provides valuable reference points for urban planners and policymakers, helping them design and implement urban greening projects more effectively.

This study evaluates the quality of sports dance teaching in a specific urban area using CNN models and deep learning technology. While the results indicate that the application of CNN models in assessing sports dance teaching quality is highly accurate and effective, the limitations of the study scope and the constraints of data samples raise questions about the generalizability of the findings. Firstly, this study focuses solely on a specific urban area as its research subject. This selection may limit the applicability of the model to other cities or regions. Different cities and regions exhibit significant differences in sports dance teaching methods, student proficiency levels, and cultural backgrounds, which affect the model's performance. Therefore, future research considers collecting data and testing models in diverse cities and regions to ensure broad applicability of the results. Secondly, the study uses a relatively limited number of images, which may impact the model's generalization ability. Deep learning models typically require large training datasets to enhance their generalization and robustness. Hence, future research expands the dataset scale, encompassing images from various backgrounds, lighting conditions, and dance movements to improve the model's performance in different contexts. Nevertheless, this study introduces a novel and effective approach for assessing sports dance teaching quality using CNN models and deep learning technology. The introduction of CNN models and deep learning technology enables more objective and accurate assessment of teaching quality. However, to ensure the broad applicability and reliability of the results, future studies validate and refine the proposed methods on a broader geographical scale and with larger datasets. While the results of this study have certain limitations, they provide valuable insights for future research. The study suggests that future studies should conduct research in different cities and regions, with an expanded dataset, to further validate and improve the proposed methods.

In addition, IoT technology has brought revolutionary changes to urban planning, but it also comes with challenges and issues. Firstly, IoT enhances the precision and efficiency of urban planning through real-time data collection and monitoring. Sensors and monitoring devices capture crucial information such as traffic flow and environmental quality, providing decision support for planners. However, this advancement raises concerns about privacy and security. With the involvement of vast amounts of personal and sensitive data, protecting user privacy and data security becomes a critical issue. This necessitates urban planners to establish strict data protection measures and security protocols when applying IoT technology. Secondly, city policies play a crucial role in the adoption and application of IoT technology. Policy support can foster the development and deployment of technology, but it must also address technical standards, regulations, and ethical issues. City policymakers need to strike a balance between encouraging innovation and protecting citizen rights. Furthermore, the complexity and cost of IoT technology pose challenges for urban planners. The procurement, deployment, maintenance, and data management of devices require substantial investment, involving economic costs and technical complexities. Urban planners need to weigh costs and benefits to ensure the feasibility and sustainability of the technology. Lastly, data processing and storage are indispensable aspects of IoT application. Managing and analyzing large volumes of data effectively is essential for extracting valuable insights. However, data management and analysis require specialized technical knowledge, posing a significant challenge for urban planners. Standardizing and interoperating data across different systems and platforms is also crucial to ensure seamless integration. In summary, the application of IoT technology in urban planning holds tremendous potential but requires comprehensive consideration and careful planning in aspects such as privacy protection, policy support, technical costs, and data processing. This study maximizes the advantages of IoT technology to continuously optimize and improve urban planning while mitigating potential risks and challenges.

## 6 Conclusion

To investigate the urban street greening planning, this study focuses on the significance of street spaces and categorizes them into four types: traffic-oriented, landscape-style, historical streets, and life leisure spaces. Using the FCN-8s image semantic segmentation framework and Baidu Street View images combined with IoT technology, the study evaluates the GLR of street spaces in the historical urban area of T City. Findings from a survey on GLR satisfaction levels through questionnaires reveal the following insights: (1) The lower the satisfaction score from street view images, the fewer corresponding images are rated satisfactory, indicating dissatisfaction with street landscape greening. Consequently, there is a need for improvement in GLR for street spaces in T City's historical urban area. (2) Statistical significance (p = 0.000 < 0.05, R^2^ = 0.919) highlights a strong positive correlation between average GLR of street spaces in T City's historical urban area and satisfaction scores of street view images. (3) Across the four types of street spaces, ranging from most to least favorable in terms of green-looking environment construction: life leisure spaces, historical streets, traffic-oriented streets, and landscape-style streets, with dissatisfaction rates of 35 %, 33 %, 20 %, and 18 %, respectively. Strategies proposed include augmenting greenery in “blind spots” and “dead corners” of street space greening through the installation of planting beds and boxes. Enhancements in street greening policies and plans aim to expand urban green spaces and integrate functional zones, promoting scientifically informed greening of urban streets. Nonetheless, research gaps remain, particularly regarding the reliance on street view images as GLR data sources, which may introduce discrepancies compared to human visual perceptions in real-world environments. Future studies should address these limitations and leverage advancements in streetscape technology for more accurate results. Additionally, further investigation and refinement are essential to overcome current constraints in IoT technology application for street space planning.

## Data availability statement

Data will be made available on request.

## CRediT authorship contribution statement

**Lei Song:** Writing – original draft, Visualization, Validation, Software, Resources, Methodology, Formal analysis, Data curation, Conceptualization.

## Declaration of competing interest

The authors declare that they have no known competing financial interests or personal relationships that could have appeared to influence the work reported in this paper.
