# Supplementary material for: The street space planning and design of artificial intelligence-assisted deep learning neural network in the Internet of Things
Source: Heliyon. 2024 Jul 22;10(15):e35031. doi: 10.1016/j.heliyon.2024.e35031 (PMC11320447; doi:10.1016/j.heliyon.2024.e35031)
Supplement: Multimedia component 1 [file mmc1.docx]

**Survey on Green Looking Ratio Satisfaction of Street Space in the Historical Urban Area of City T**

Hello! Thank you very much for participating in this survey! This questionnaire aims to understand the opinions of primary school physical education teachers regarding physical education homework. The survey is conducted anonymously, and the collected data will be kept confidential, for academic research purposes only. We hope you fill it out based on your actual experiences. Please mark "√" on the appropriate options or fill in the blanks. Once again, we appreciate your support!

I. Basic Information

1. Your residence (activity) area:

A. Within the historical district of City T

B. Other regions

2. Gender:

A. Male

B. Female

3. Age:

A. 18 years and below

B. 19-30 years

C. 31-45 years

D. 46-55 years

E. 56 years and above

II. Please observe the following street images. Based on your visual perception, choose the corresponding satisfaction level of the green visual experience. (Focus only on the green visual experience rating of the street, without paying too much attention to the overall landscape aesthetics. Very poor = -2 points, Poor = -1 point, Average = 0 points, Good = 1 point, Very good = 2 points):


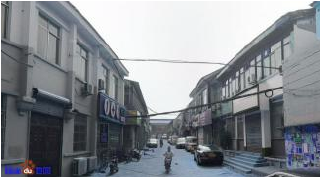
 Rating for Figure 1:


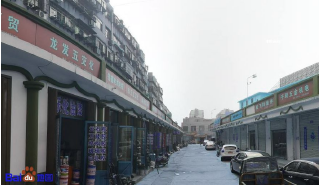
 Rating for Figure 2:


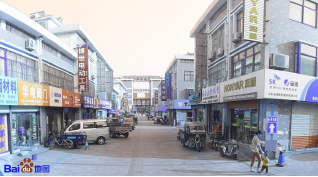
 Rating for Figure 3:


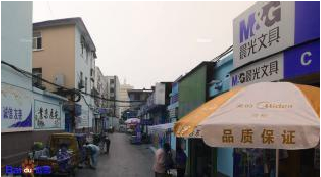
 Rating for Figure 4:


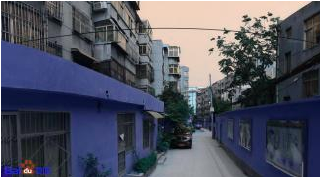
 Rating for Figure 5:


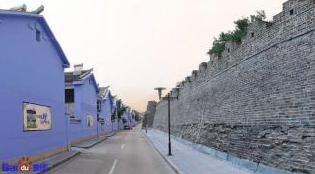
 Rating for Figure 6:


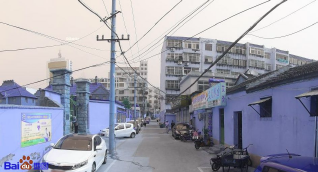
 Rating for Figure 7:


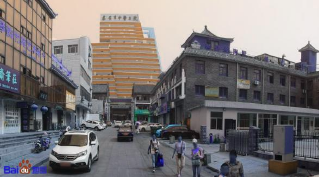
 Rating for Figure 8:


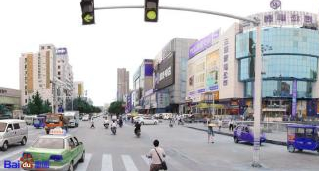
 Rating for Figure 9:


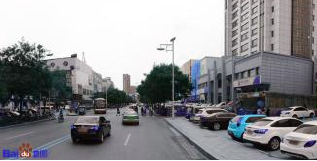
 Rating for Figure 10:


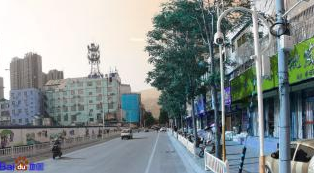
 Rating for Figure 11:


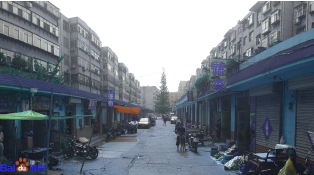
 Rating for Figure 12:


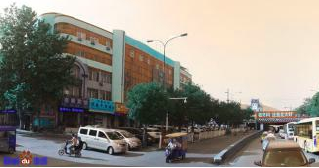
 Rating for Figure 13:


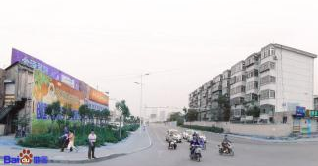
 Rating for Figure 14:


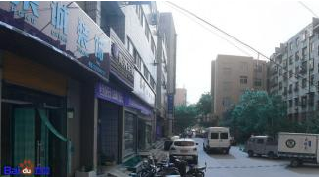
 Rating for Figure 15:


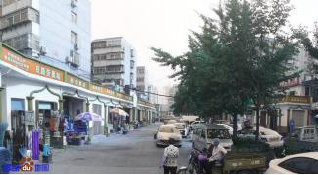
 Rating for Figure 16:


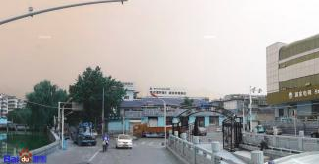
 Rating for Figure 17:


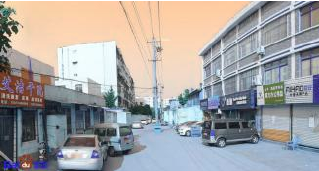
 Rating for Figure 18:


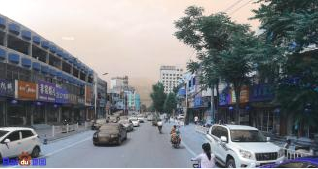
 Rating for Figure 19:


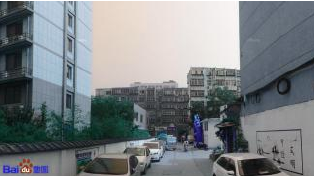
 Rating for Figure 20:


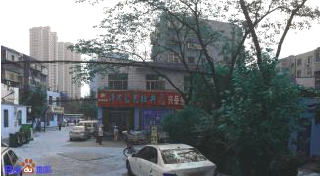
 Rating for Figure 21:


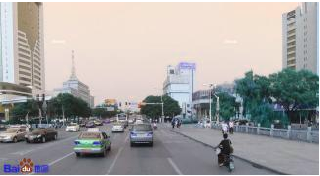
 Rating for Figure 22:


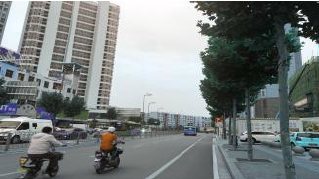
 Rating for Figure 23:


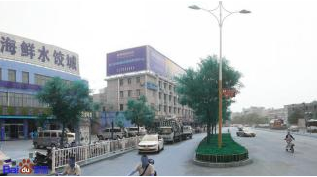
 Rating for Figure 24:


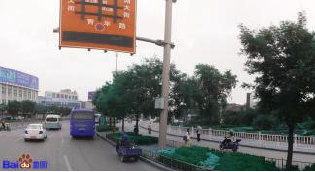
 Rating for Figure 25:


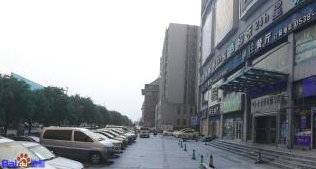
 Rating for Figure 26:


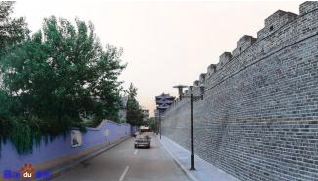
 Rating for Figure 27:


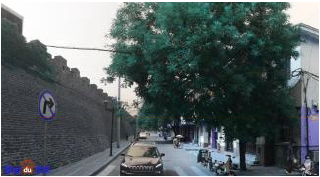
 Rating for Figure 28:


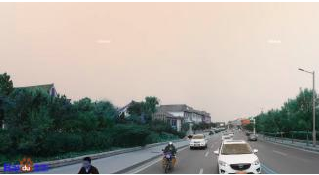
 Rating for Figure 29:


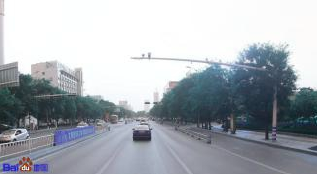
 Rating for Figure 30:


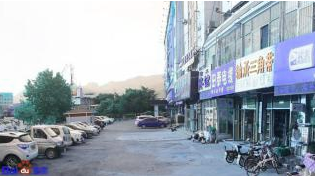
 Rating for Figure 31:


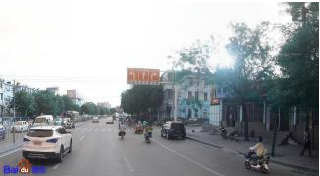
 Rating for Figure 32:


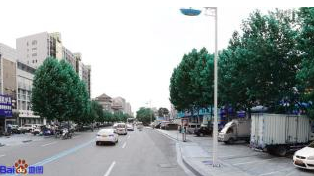
 Rating for Figure 33:


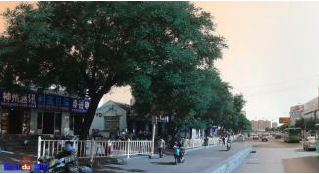
 Rating for Figure 34:


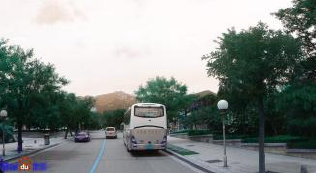
 Rating for Figure 35:


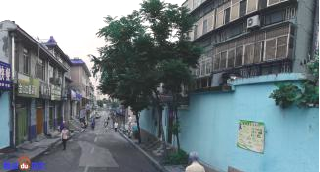
 Rating for Figure 36:


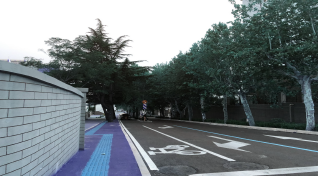
 Rating for Figure 37:


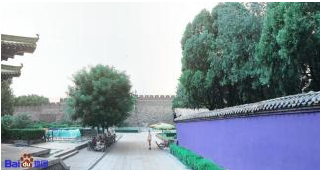
 Rating for Figure 38:


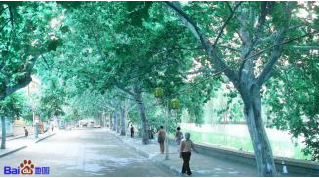
 Rating for Figure 39:


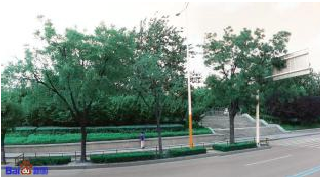
 Rating for Figure 40:


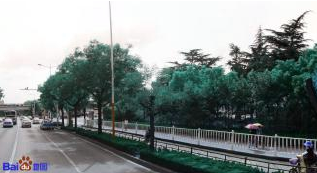
 Rating for Figure 41:


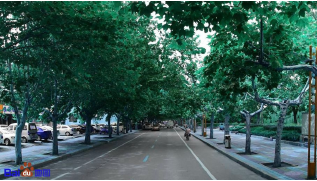
 Rating for Figure 42:


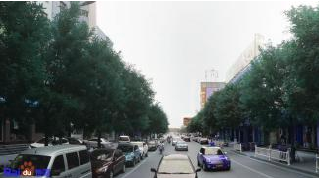
 Rating for Figure 43:


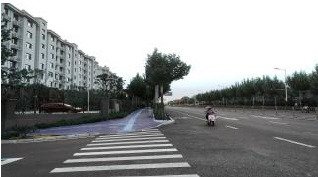
 Rating for Figure 44:


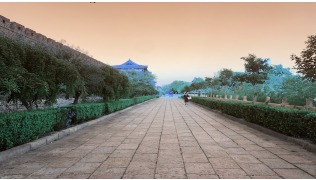
 Rating for Figure 45:


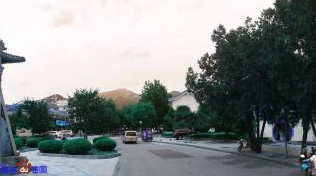
 Rating for Figure 46:


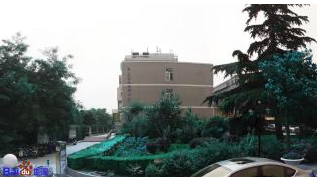
 Rating for Figure 47:


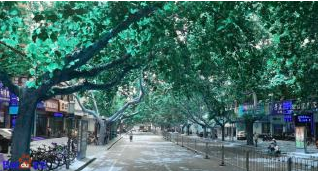
 Rating for Figure 48:

The questionnaire concludes here. Thank you very much for your cooperation. Wishing you a fulfilling life and smooth work!
